# Activation and Catalysis of Methane over Metal–Organic Framework Materials

**DOI:** 10.1021/accountsmr.4c00279

**Published:** 2024-11-07

**Authors:** Bing An, Yujie Ma, Xue Han, Martin Schröder, Sihai Yang

**Affiliations:** † Department of Chemistry, University of Manchester, Manchester M13 9PL, U.K.; ‡ College of Chemistry, Beijing Normal University, Beijing 100875, China; § College of Chemistry and Molecular Engineering, Beijing National Laboratory for Molecular Sciences, Peking University, Beijing 100871, China

## Abstract

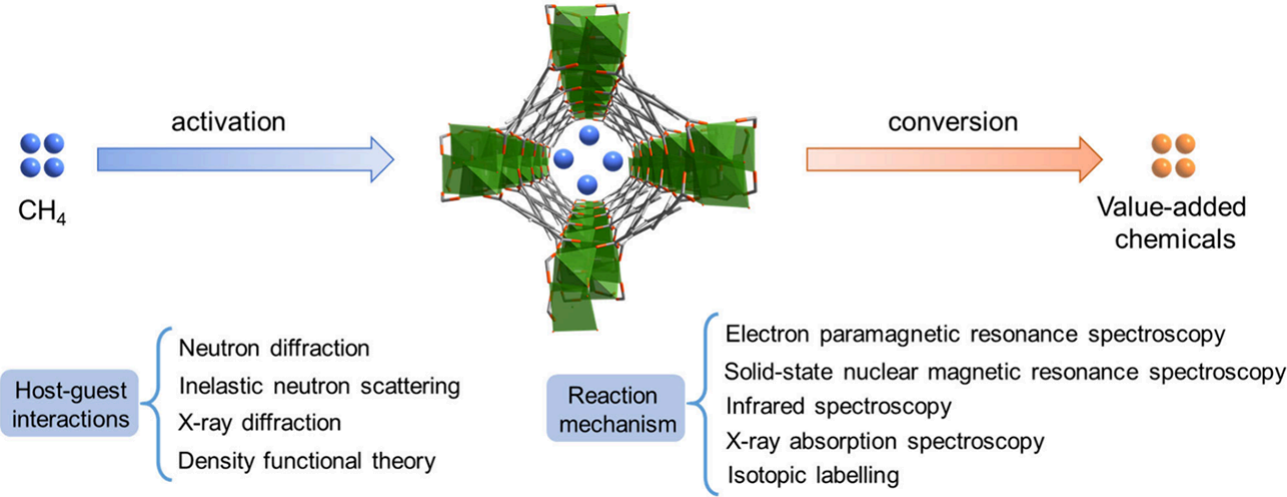

Methane (CH_4_), which is the main component of natural gas, is an abundant and widely available carbon resource. However, CH_4_ has a low energy density of only 36 kJ L^–1^ under ambient conditions, which is significantly lower than that of gasoline (*ca*. 34 MJ L^–1^). The activation and catalytic conversion of CH_4_ into value-added chemicals [*e.g*., methanol (CH_3_OH), which has an energy density of *ca*. 17 MJ L^–1^], can effectively lift its energy density. However, this conversion is highly challenging due to the inert nature of CH_4_, characterized by its strong C–H bonds and high stability. Consequently, the development of efficient materials that can optimize the binding and activation pathway of CH_4_ with control of product selectivity has attracted considerable recent interest. Metal–organic framework (MOF) materials have emerged as particularly attractive candidates for the development of efficient sorbents and heterogeneous catalysts due to their high porosity, low density, high surface area and structural versatility. These properties enable MOFs to act as effective platforms for the adsorption, binding and catalytic conversion of CH_4_ into valuable chemicals. Recent reports have highlighted MOFs as promising materials for these applications, leading to new insights into the structure–activity relationships that govern their performance in various systems.

In this Account, we present analysis of state-of-the-art MOF-based sorbents and catalysts, particularly focusing on materials that incorporate well-defined active sites within confined space. The precise control of these active sites and their surrounding microenvironment is crucial as it directly influences the efficiency of CH_4_ activation and the selectivity of the resulting chemical products. Our discussion covers key reactions involving CH_4_, including its activation, selective oxidation of CH_4_ to CH_3_OH, dry reforming of CH_4_, nonoxidative coupling of CH_4_, and borylation of CH_4_. We analyze the role of active sites and their microenvironment in the binding and activation of CH_4_ using a wide range of experimental and computational studies, including neutron diffraction, inelastic neutron scattering, and electron paramagnetic resonance, solid-state nuclear magnetic resonance, infrared and X-ray absorption spectroscopies coupled to density functional theory calculations. In particular, neutron scattering has notable advantages in elucidating host–guest interactions and the mechanisms of the conversion and catalysis of CH_4_ and CD_4_. In addition to exploring current advances, the limitations and future direction of research in this area are also discussed. Key challenges include improvements in the stability, scalability, and performance of MOFs under practical conditions, as well as achieving higher selectivity and yields of targeted products. The ongoing development of MOFs and related materials holds great promise for the efficient and sustainable utilization of CH_4_, transforming it from a low-density energy source into a versatile precursor for a wide range of value-added chemicals. This Account summarizes the design and development of functional MOF and related materials for the adsorption and conversion of CH_4_.

## 1 Introduction

Methane (CH_4_) is the main component of natural gas and is extracted from natural gas wells and sweetened into a consumer-grade gas stream. This is then piped to distributors and consumers, and in most cases, burned directly for heat. With the depletion of oil resources, there has been considerable interest in converting CH_4_ into value-added products, serving as an important alternative to the oil-based production of fuels and chemicals.^1^ However, harsh conditions (high pressure and/or temperature) are often required to bind and activate CH_4_ due to its intrinsic inertness, but once activated CH_4_ is oxidized very readily to carbon dioxide (CO_2_) due to the low energy barrier between the intermediate products and CO_2_. Thus, the main challenges in this area are to bind and activate CH_4_ efficiently under mild conditions and to increase the selectivity for intermediate products rather than CO_2_.^2^ Several strategies have been developed in recent years to address these issues, for example, reducing or removing the oxidant during the activation of CH_4_ to prevent its overoxidation; using softer transformation agents (e.g., Cu–O clusters)^3^ in CH_4_ borylation and oxidation; designing catalysts with isolated sites for CH_4_ activation and product formation. Functional porous metal–organic frameworks (MOFs) have been extensively investigated as sorbents for adsorption and conversion of CH_4_ due to their capacity to integrate M-O clusters with binding and catalytic sites and mild oxidation reagents within their framework structures.^4^ In this Account, we highlight our recent research advances in the development of MOFs with well-defined active sites for CH_4_ activation and catalysis. This work will contribute to the design of improved MOF-based catalytic systems for future CH_4_ utilization.

## 2 Adsorption of CH_4_

The adsorption of CH_4_ can enable its activation and catalytic conversion by interaction with active sites on the surface. In this way adsorption can facilitate the activation of the strong C–H bonds in CH_4_ and thus promote the conversion of CH_4_ to valuable chemicals or fuels. In addition, efficient adsorption ensures that CH_4_ molecules are held in close proximity to the catalytic sites, enhancing the overall rate of reaction and selectivity. The optimization of the adsorption properties of a catalyst is thus essential for improving the efficiency and effectiveness of CH_4_ conversion processes.

Adsorption of CH_4_ by MOFs has gained very significant interest due to the high porosity and facile immobilization of active sites within MOFs.^5,6^ The designable nature of MOFs allows fine-tuning of their structures and properties to enhance host–guest interactions, thereby improving adsorption performance. Here we summarize the studies on CH_4_ binding in functional MOFs, particularly highlighting the role of host–guest interactions. Functional groups play a crucial role in polarization, framework flexibility, and chemical affinity of MOFs. Several studies have demonstrated the effectiveness of ligand functionalization in enhancing adsorption performance in MOFs.

**Figure 1:**
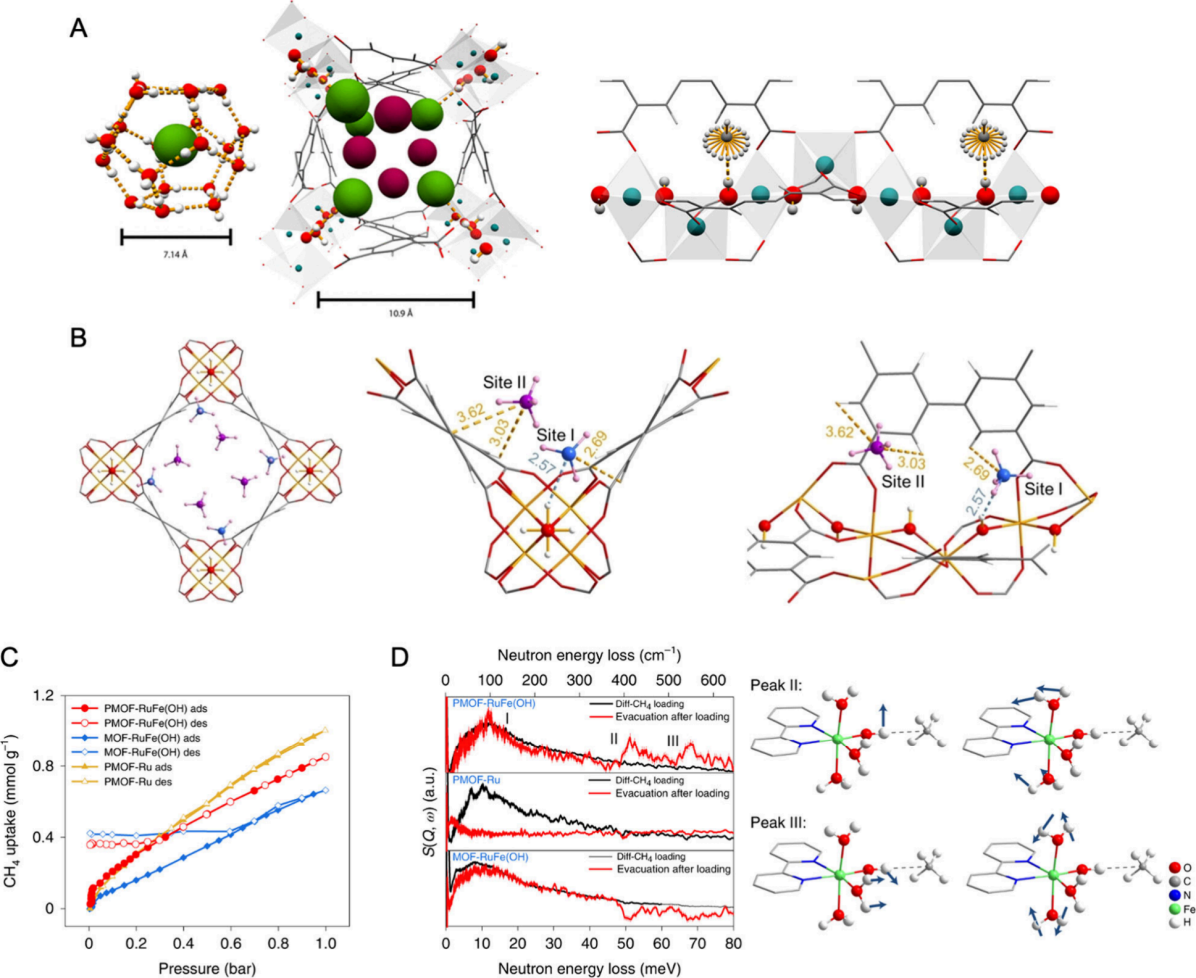
Host–guest interactions between CH_4_ molecules and hydroxyl groups within MOFs. (A,B) M-(μ_2_-)OH sites in MFM-300(In) and MFM-300(Fe) for CH_4_ binding. (A) Cage structure and H_3_C–H^guest^···O^host^ interaction of CH_4_ in a clathrate (left); and channel and H_4_C^guest^···H–O^host^ interaction of CH_4_ in MFM-300(In) (Site I: green, 0.80 CD_4_ per In site; Site II: purple, 0.29 CD_4_ per In site) (middle). View of the structural model showing the almost free rotation of CH_4_ molecules at the bridging hydroxyl group (right). All structures have been obtained by Rietveld refinement of neutron powder diffraction (NPD) data (C, gray; O, red; In, blue; H, white).^10^ (B) Crystal structures of CD_4_-loaded MFM-300(Fe) after degassing, with around 0.14 CD_4_ per Fe site (Site I: 0.07 CD_4_ per Fe; Site II: 0.07 CD_4_ per Fe) remaining trapped within the structure. All structures are derived from Rietveld refinement of NPD data (C, gray; O, red; Fe, light orange; D, pink; H, white; the C atoms of CD_4_ are labeled by different colors for clarity). (C,D) Fe-(μ-)OH sites in PMOF-RuFe(OH) for CH_4_ binding. (C) Adsorption (ads)/desorption (des) isotherms for CH_4_ in PMOF-RuFe(OH), PMOF-Ru and MOF-RuFe(OH) at 298 K. (D) Comparison of the difference (diff) INS spectra of bare and CH_4_-loaded sample and the difference INS spectra on evacuation for PMOF-RuFe(OH), PMOF-Ru and MOF-RuFe(OH) (left). Vibrational modes of peaks II and III are shown on the right. For both PMOF-RuFe(OH) and MOF-RuFe(OH), some residual CH_4_ remains on desorption, whereas PMOF-Ru shows complete desorption. The binding structure has been optimized by DFT calculations based on INS and FTIR spectra. Reproduced with permission from refs (9), (10), and (12). Copyright 2016 American Chemical Society, Copyright 2024 The Authors, and Copyright 2022 The Author(s), under exclusive license to Springer Nature Limited.

The materials MFM-300(M), [M_2_(OH)_2_(L)] (M = trivalent metal ion; L = 3,3′,5,5′-biphenyltetracarboxylate, bptc^4–^) are a series of isostructural MOFs assembled from one-dimensional *cis*-[MO_4_(OH)_2_] chains linked by μ_2_–OH groups and bptc^4–^ ligands.^7−9^ MFM-300(In) shows promise for CH_4_ adsorption due to its high volumetric uptake and specific host–guest interactions.^9^ The binding and rotation of CH_4_ within MFM-300(In) have been investigated using neutron powder diffraction (NPD) and inelastic neutron scattering (INS). NPD allows the direct visualization of structural details of adsorption sites, while INS reveals the binding dynamics of host-guest interactions (see Section S1 for further details). Coupled with density functional theory (DFT) modeling, these studies have revealed the primary binding site for CH_4_ in MFM-300(In) to be at the bridging hydroxyl group within the pores via H_3_C–H^guest^···O^host^ interactions (Figure 1A). Interestingly, this interaction is distinct from the binding observed in CH_4_/water clathrates, where CH_4_ is trapped within water cages without specific binding to hydroxyl groups. The host–guest interactions in CH_4_@MFM-300(In) are further supplemented by supramolecular contacts between CH_4_ and aromatic rings, collectively contributing to the high efficiency of CH_4_ binding. The adsorbed CH_4_ molecules display weakly hindered rotational motion, even at low temperatures, indicating a relatively free rotation within the pore. The study highlighted that adsorbed CH_4_ molecules in MFM-300(In) packed very efficiently within the pores, with densities comparable to those of liquid CH_4_. This efficient packing model is attributed to a combination of geometric confinement by the MOF structure and specific host–guest interactions. Compared with MFM-300(In), MFM-300(Fe) shows stronger binding interactions to CH_4_, enabling exceptional adsorption of CH_4_ at low pressures.^10^ At 1 mbar and 298 K, MFM-300(Fe) shows a superior CH_4_ uptake of 0.85 mmol g^–1^, representing a benchmark for the capture of CH_4_ at low pressures. In addition, the efficient separation of CH_4_/N_2_ can be achieved with MFM-300(Fe), as confirmed by dynamic breakthrough experiments. Complementary experimental and modeling methods reveal that the host–guest binding interactions between CH_4_ molecules and MFM-300(Fe), including {Fe–OH···CH_4_} and {C···phenyl ring} interactions, are key to the exceptional adsorption of CH_4_ at low pressures (Figure 1B).

The basicity of binding sites significantly influences the CH_4_ adsorption performance. Compared with μ_2_–OH, the less acidic μ–OH group (*i.e.,* more basic) derived from coordination to a single metal center can show enhanced CH_4_ adsorption due to its enhanced electron-donating ability. CH_4_ adsorption and conversion in MOFs can be enhanced by incorporating iron-hydroxyl sites, mimicking the function of methane monooxygenase.^11^ We integrated a monoiron hydroxyl site [Fe–OH], with a redox-active polyvanadotungstate and photosensitizer within a MOF scafford (UiO-67) to afford PMOF-RuFe(OH).^12^ The CH_4_ isotherms of PMOF-RuFe(OH) display a steep initial rise and a significant residual uptake upon desorption under a vacuum at room temperature (*ca*. 0.5 CH_4_/Fe). This behavior is not observed for the isostructural MOF, PMOF-Ru, which does not incorporate the active Fe–OH site (Figure 1C). INS and DFT simulations reveal that the interaction between [(bpy)Fe(OH)(H_2_O)_3_]^2+^ and CH_4_ molecules is predominantly driven by strong hydrogen bonding (Fe–OH···CH_4_ = 2.39 Å) (Figure 1D). The unique [Fe–OH] sites within the MOF facilitated strong binding of CH_4_, thus lowering the barrier for C–H bond activation and enabling efficient conversion to CH_3_OH. These findings highlight the importance of hydroxyl groups in designing efficient materials for CH_4_ adsorption and activation. In addition to hydroxyl sites, other functional groups can also enhance CH_4_ adsorption by improving the interactions between the framework and CH_4_ molecules. For example, UTSA-76 featuring pyrimidine groups has been reported to improve CH_4_ binding and storage capacity.^13^

**Figure 2:**
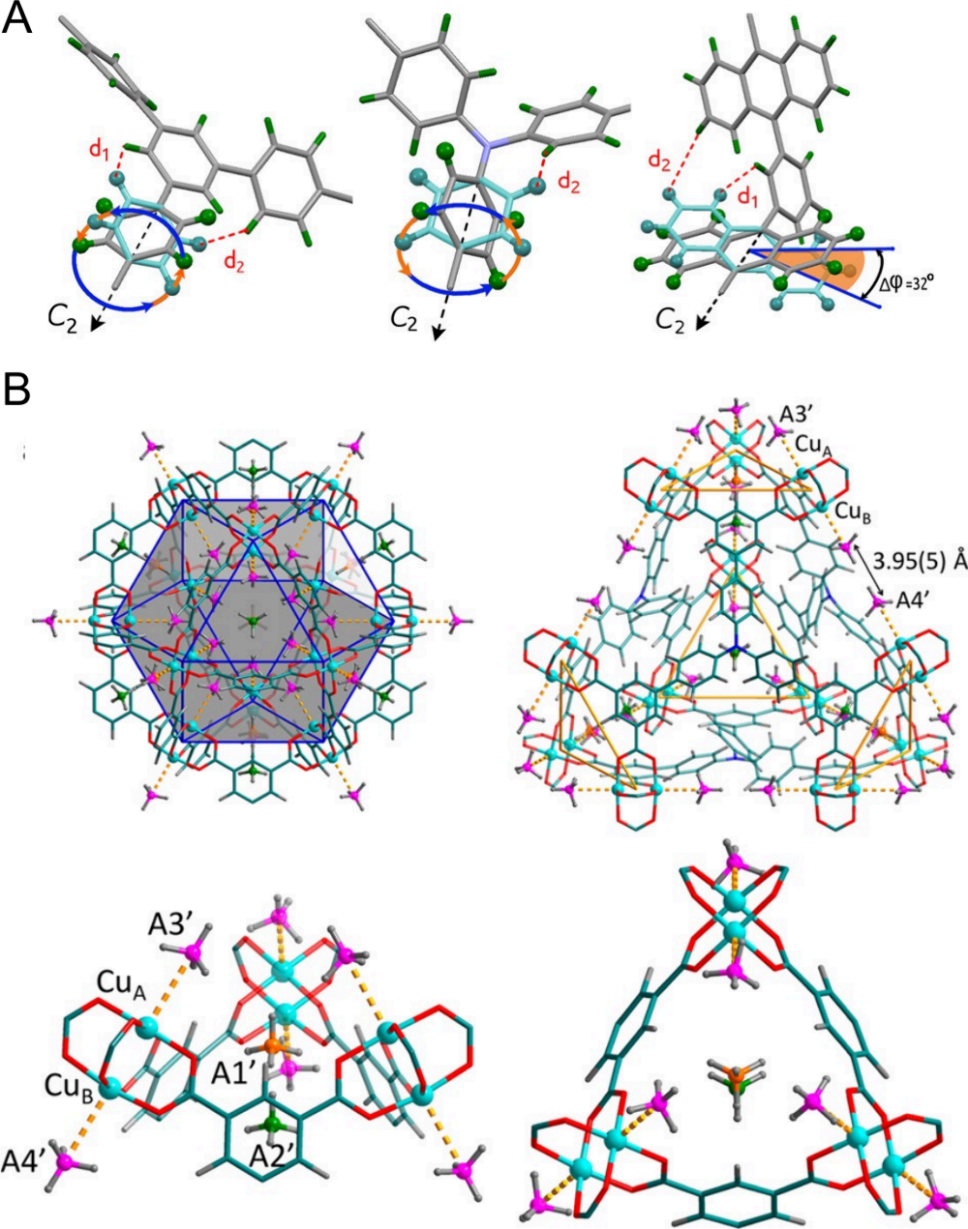
Optimal molecular dynamics lead to exceptional CH_4_ uptake. (A) Rotational models of MFM-112, MFM-115, and MFM-132. (B) CD_4_ adsorption sites in MFM-115 at a loading of 1.0 CD_4_/Cu. Sites **A1′** and **A2′** are found within the triangular [(Cu_2_)_3_(isophthalate)_3_] window, and **A3′** and **A4′** are located on the Cu(II) sites. C, teal; H, gray; Cu, aqua; site **A1′**, orange; site **A2′**, green; sites **A3′** and **A4′**, pink. Reproduced with permission from ref (14). Copyright 2017 American Chemical Society.

In addition to binding sites provided by functional groups, vacant metal centers and confinement within pore environments are also important to generate binding sites for guest molecules. For example, MFM-112, MFM-115 and MFM-132 with different bridging ligands show different CH_4_ uptake capacities. MFM-115 demonstrates an exceptionally high deliverable CH_4_ capacity of 208 v/v between 5 and 80 bar at room temperature, outperforming other high-performing MOFs.^14^ The primary binding sites for CD_4_ were located within the small pockets enclosed by the [(Cu_2_)_3_(isophthalate)_3_] window and three anthracene/phenyl panels as determined by NPD studies (Figure 2). Thus, the vacant Cu(II) sites are only secondary or tertiary adsorption sites in these structures. Thus, a tight hydrophobic cavity can generate stronger binding affinity for CH_4_ than vacant metal sites, and this structural configuration allowed optimal molecular dynamics and pore geometry to enable the observed enhanced CH_4_ uptake. Similarly, a bismuth-based MOF, NOTT-220 (or MFM-220), exhibits strong binding sites [Bi–O···H–CH_3_] for CH_4_, significantly enhancing its adsorption capacity, with a CH_4_ uptake of 287 V/V at 20 bar and 195 K.^15^ These studies emphasize the role of metal centers and linker interactions in creating an appropriate environment for CH_4_ adsorption. In addition, flexible MOFs with dynamic structures, such as MOF-520 and Co(bdp) (bdp^2–^ = 1,4-benzenedipyrazolate), also exhibit tailored adsorption properties under varying pressure and temperature conditions.^16,17^

In summary, structural features such as the pore geometry, functional groups, and specific binding sites significantly influence the adsorption capacity and characteristics of these materials. By advancing our understanding of host–guest dynamics, MOFs can be further developed to address critical environmental challenges, including CH_4_ capture and utilization. The integration of catalytic functionalities within MOFs represents an exciting frontier and promising new avenue for the production of sustainable energy and value-added chemicals.

## 3 Conversion of CH_4_

### 3.1 Milestones in the Conversion of CH_4_

**Figure 3:**
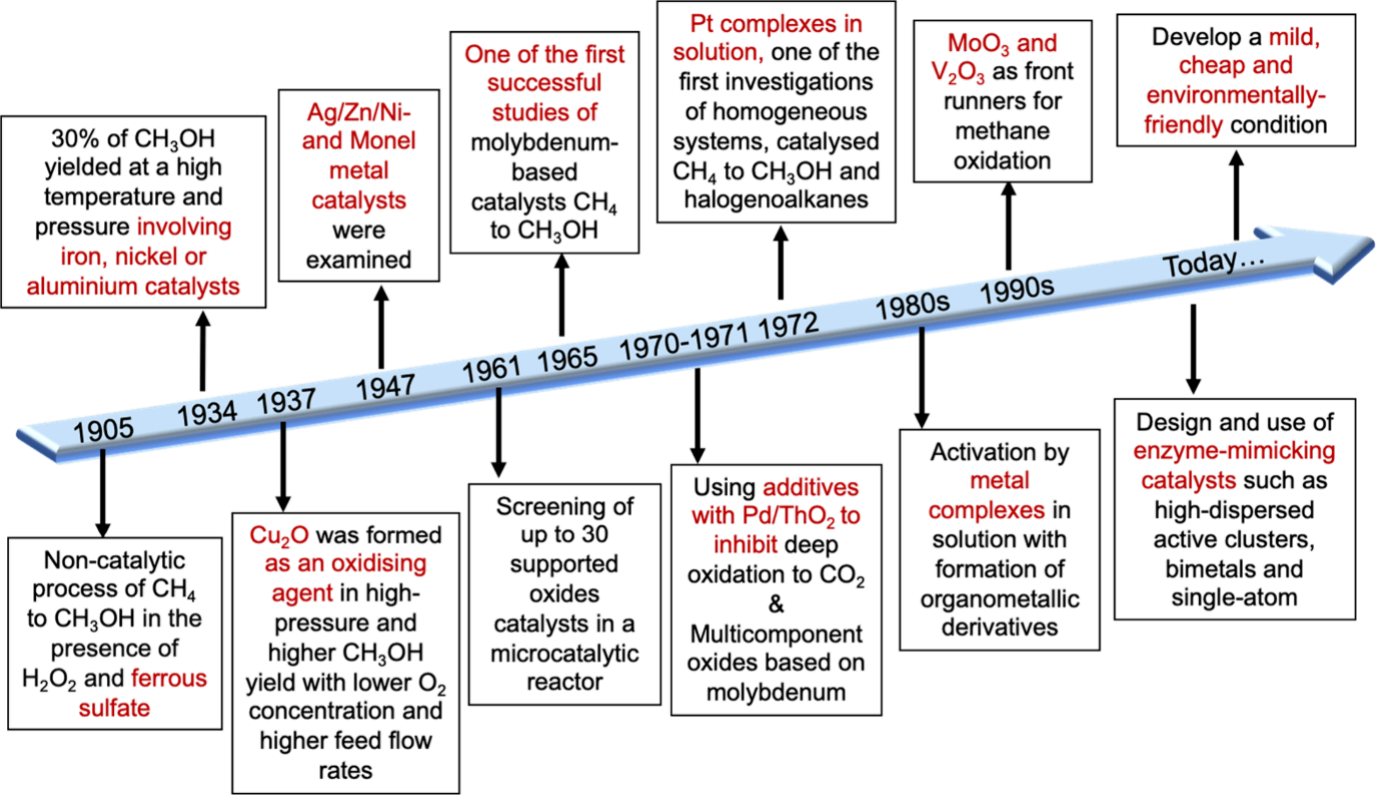
Timeline for selected research milestones in CH_4_ conversion.

Research into the oxidation of CH_4_ dates back to the early 20th century. The timeline shown in Figure 3 illustrates the key developments in methane to methanol conversion catalysis and highlights advances in catalyst materials and processes, including the use of metal complexes, supported oxides, and enzyme-mimics to improve efficiency and sustainability. A great deal of effort has been devoted to functionalizing C–H bonds in methane. To date, highly active and stable catalysts that enable CH_4_ conversion under mild conditions include enzyme mimics, highly dispersed clusters, bimetals, and single atom catalysts. Moreover, partial oxidation of CH_4_ to CH_3_OH, dry reforming of CH_4_ to hydrocarbons, and borylation of CH_4_ have emerged as important areas of research to address the energy crisis, and the products can be used in further downstream reactions. Considering their scientific and industrial significance, research advancements, and practical applications in this field, selected topics on CH_4_ activation are discussed below.

### 3.2 Partial Oxidation of CH_4_ to CH_3_OH

**Figure 4:**
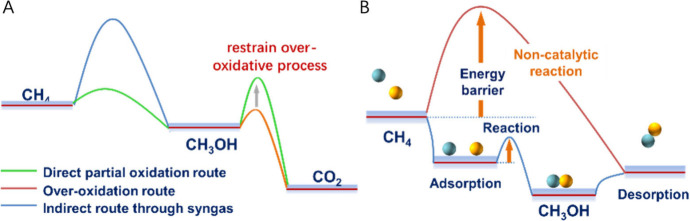
Schematic view of energy barriers to oxidation of CH_4_ to CH_3_OH: (A) partial oxidation of CH_4_ through syngas and direct route to CH_3_OH which is highly susceptible to oxidation to CO_2_; (B) comparison of noncatalytic conversion (orange) and the catalysis *via* lowering of the energy barrier (blue).

Over 110 million tonnes of CH_3_OH are produced each year from syngas (CO/H_2_), which is typically produced by steam reforming [CH_4_ + H_2_O → CO + 3H_2_] or coal gasification.^18^ In comparison, direct production of CH_3_OH from CH_4_ is more environmentally friendly and financially attractive. However, it is worth noting that the C–H bond dissociation energy is around 439 kJ mol^–1^ for CH_4_ but is only 47 kJ mol^–1^ for CH_3_OH; thus, once activated, CH_4_ is prone to overoxidation to CO_2_ in preference to CH_3_OH (Figure 4). Some novel methodologies proposed to avoid overoxidation are listed in Sections S2 and S3 and pathways of MOF catalysts incorporating molecular active sites for activation of CH_4_ shown in Section S4. In this sense, it is critical to develop catalysts that can activate the C–H bond in CH_4_ but also inhibit further oxidation of CH_3_OH to CO_2_.

Research into advanced catalysts and systems for low-temperature C–H activation is essential for the direct conversion of CH_4_. Processes operating at low temperature, thermocatalytic systems, or those driven by photocatalytic and electrocatalytic routes point the way to achieving efficient CH_4_ conversion with potentially notably reduced energy costs. Recent studies have demonstrated the immense potential of MOF catalysts for selective CH_4_ oxidation *via* thermal catalysis, photocatalysis, and electrocatalysis. In this section, we summarize and discuss the latest advances in the use of MOF catalysts for the direct oxidation of CH_4_ using thermal, light, or electrical energy, with a focus on the potential of MOF catalysts for carrying out this conversion under mild conditions. State-of-the-art examples of MOF-based catalysts that have been experimentally studied for CH_4_ oxidation are summarized in Table 1.^12,19−38^

**Table 1:** Summary of Selected MOF-Catalyzed Oxidation of CH_4_ to CH_3_OH

Catalyst	Oxidant	Pressure (bar)	Temperature (°C)	Condition	CH_3_OH time yield (μmol·g_cat_^–1^·h^–1^)
Cu-NU-1000^19^	O_2_	1	200	Thermal catalysis, loop	17.7
Cu-2.9-NU-1000^20^	O_2_	1	200	Thermal catalysis, loop	1.5
Cu-2.9-NU-1000^20^	O_2_	40	200	Thermal catalysis, loop	6.5
MOF-808-Bzz-Cu^21^	N_2_O	1	150	Thermal catalysis, loop	71.8 ± 23.4
Cu/ZIF-7^22^	H_2_O_2_	30	150	Thermal catalysis, batch	44.4
Zn_*x*_Cu_1–x_(mim^–1^)_2_^23^	O_2_	1	200	Thermal catalysis, loop	0.1
MIL-53(Al,Fe)^24^	H_2_O_2_	30.5	50	Thermal catalysis, batch	450
MIL-100(Fe)^25^	N_2_O	0.015	200	Thermal catalysis, loop	227
PCN-250^26^	N_2_O	1	220	Thermal catalysis, loop	52.5
UiO-66(2.5TFA)-Fe^27^	H_2_O_2_	30	50	Thermal catalysis, batch	259
UiO-66-H^28^	H_2_O_2_	30	50	Thermal catalysis, batch	364^a^
Fe^3+^@UiO-66-C_14_^29^	H_2_O_2_	30	50	Thermal catalysis, batch	440
Fe-ZSM-5@ZIF-8^30^	H_2_O	1	150	Thermal catalysis, loop	0.24
Fe/UiO-66^31^	O_2_	5	180	Thermal catalysis, loop	7.2
Ce-UiO-Co(OH)^32^	H_2_O_2_	30	80	Thermal catalysis, batch	163
Ru_1_/UiO-66^33^	H_2_O_2_	30	70	Thermal catalysis, batch	3675
AuPd@ZIF-8^34^	H_2_O_2_+O_2_	30	50	Thermal catalysis, batch	967
AuPd@Cu-UiO-66_100_^35^	H_2_O_2_	30	70	Thermal catalysis, batch	199
UiO-67-Pt-Z^36^	H_2_O_2_	50	60	Thermal catalysis, batch	3.34^b^
PMOF-RuFe(OH)^12^	O_2_ + H_2_O	∼1	r.t.	Photocatalysis, batch	3145 ± 340
	O_2_ + H_2_O	∼1	r.t.	Photocatalysis, flow	8810 ± 340
Fe@PCN-222^37^	H_2_O_2_	20	r.t.	Photocatalysis, batch	1465
Mg-MOF-74^38^	-	15	20	Electrocatalysis, flow cell	66.1^c^/60.6^d^

a C1 oxygenates as products including CH_3_OH, CH_3_OOH, and CH_3_OOH.

b Unit: ppm.

c Formate time yield.

d Acetate time yield. r.t.= room temperature.

#### 3.2.1 Thermocatalytic Oxidation of CH_4_

**Figure 5:**
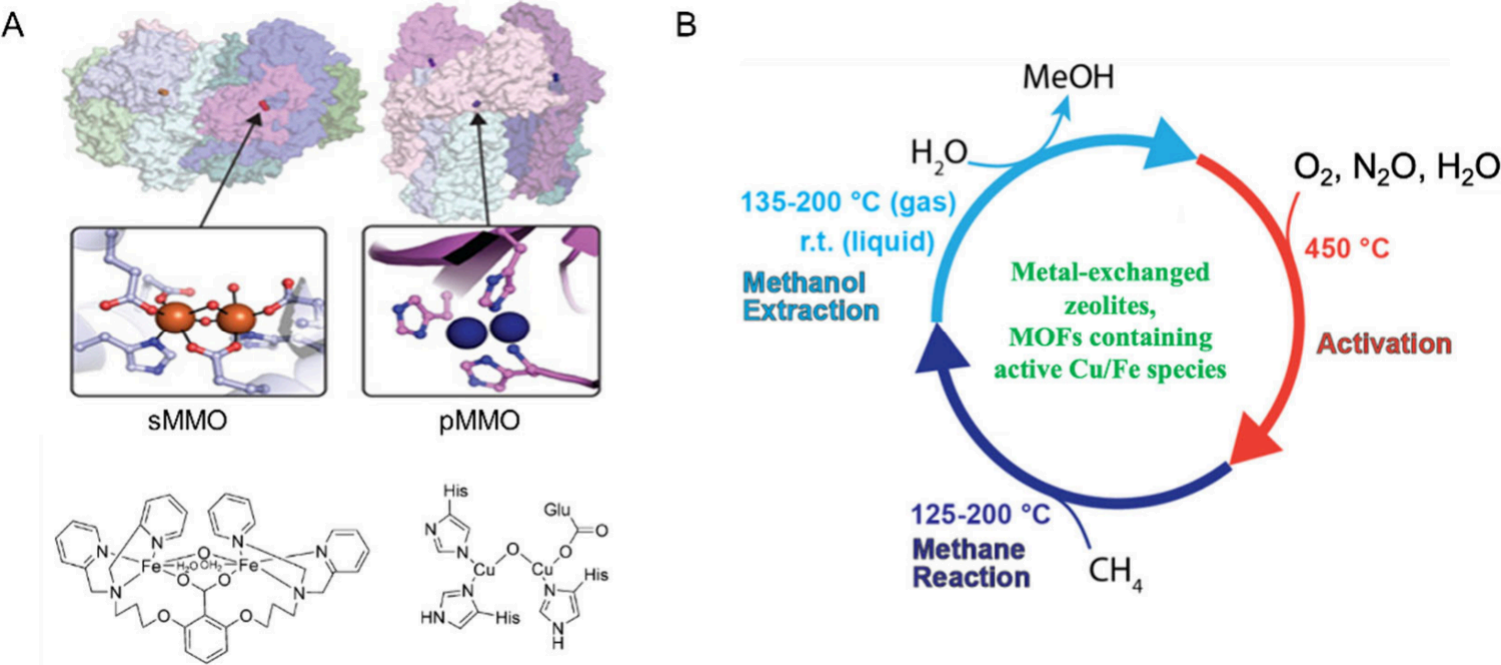
(A) Illustration of sMMO and pMMO. Left: Crystal structure of the sMMO complex from *Methylococcus capsulate* (Bath) (PDB code 4GAM) with iron sites shown in orange, α subunits shown in shades of blue, β subunits shown in teal and light cyan, γ subunits shown in green, and MMOB shown in pink and magenta (top). Right: Crystal structure of the pMMO trimer from *Methylococcus capsulatus* (Bath) (PDB 3RGB) with copper ions shown as blue spheres, PmoB subunits shown in shades of purple and pink, PmoA subunits shown in shades of blue, and PmoC subunits shown in shades of teal and cyan. The active site of pMMO is shown modeled as a dicopper center (bottom). (B) Illustration of the three-step conversion of CH_4_ to CH_3_OH including (i) typical activation of the material by oxidants, (ii) oxidation of CH_4_ to form surface-bound precursors to CH_3_OH, and (iii) extraction of CH_3_OH, for example, with water. Reproduced with permission from refs (2) and (40). Copyright 2017 American Chemical Society and 2016 Elsevier.

Partial oxidation of CH_4_ to CH_3_OH usually operates at relatively high pressures (20–100 bar) and temperatures (350–500 °C), but the formation of superoxide radical (O_2_^•-^) usually results in the complete oxidation of CH_4_ to CO_2_. Recently, methanotrophic bacteria, denoted as “methane monooxygenases (MMOs)”, have been discovered as two different types: soluble-MMOs (sMMO) and particulate-MMOs (pMMO).^39−41^ sMMO contains a diiron active center and dinuclear Fe(IV) intermediate cluster, while pMMO contains a dicopper center with copper(I/II) atoms having a single set of O/N ligands (Figure 5A). They both utilize CH_4_ as the sole energy source and oxidize CH_4_ to CH_3_OH in the first step of metabolism of CH_4_. This has inspired the development of selective CH_4_ conversion processes using catalysts that mimic MMOs. For example, Fe- and Cu-exchanged zeolites have been studied widely for CH_4_ oxidation *via* a 3-step cyclic gas-phase oxidation process with O_2_, N_2_O or H_2_O. This involves the activation of the catalysts by oxidants and the desorption of the produced CH_3_OH (Figure 5B).^2,42^ However, the chemical structure of these active sites has not been determined precisely in these cation-exchanged zeolite catalysts due to their lack of structural and chemical order.

MOF-based catalysts offer unique advantages in the characterization of the active sites and catalytic mechanisms due to their structures and high crystallinity. For example, mononuclear, dinuclear and trinuclear copper centers have been incorporated into the NU-1000 framework to facilitate the transformation of CH_4_ into CH_3_OH.^19,20^ Similarly, designing and subsequently altering these dicopper centers within MOF catalysts have unveiled their exceptional ability for selective CH_4_ oxidation to CH_3_OH.^21^ Monocopper centers in ZIFs also show activity in CH_4_ oxidation to CH_3_OH.^22,23^ In contrast to pMMO, where the precise active site is still under debate, the active site of sMMOs has been identified as a dimeric Fe active site.^40,41^ Several MOFs with iron active sites that mimic enzymatic sites have been investigated for oxidation of CH_4_,^24−31^ also with the aim of addressing ambiguity around the active sites in biobased systems. Oxidative intermediates, *e.g*., metal-oxo species, act as highly reactive intermediates capable of abstracting hydrogen from CH_4_, forming methyl radicals that produce CH_3_OH. By confining active sites in MOFs, the reactivity is limited to the immediate vicinity of the active site, thus hindering further oxidation of the CH_3_OH that is produced.

It is well established that organometallic complexes with unique electronic properties also exhibit the ability to activate C–H bonds. For example, Pt complexes [*e.g*., Pt-NHC, Pt(PMe_3_)_4_] possessing high electron density and high electrophilicity (the ability to attract electrons) can interact and weaken the C–H bond.^36^ In addition, electron-poor d^0^ transition metal alkyls and hydrides (*e.g*., Cp_2_ZrH_2_, Cp*Sc(CH_3_)_2_) can also activate the C–H bond of CH_4_ through σ-bond metathesis.^43^ MOFs, exploiting the tunability of the design of metal oxide nodes and ligands, can replicate similar active metal sites in the structures, which not only enhances the stability of the catalysts but also improves their selectivity and activity.^32−36^ In addition, MOFs can stabilize solution-inaccessible catalytic species *via* active-site isolation, providing a blueprint for the design and generation of new catalytic sites for CH_4_ oxidation. For example, [Ce-UiO-Co(OH)] can undergo σ-bond metathesis with the C–H bond of CH_4_ to facilitate activation and subsequent conversion to CH_3_OH with a high turnover number (TON) of 3250.^32^ Nevertheless, to date, studies on MOF catalysis for CH_4_ conversion remain relatively scarce.

#### 3.2.2 Photocatalytic Oxidation of CH_4_

Compared with thermal catalytic processes, photocatalytic conversion of CH_4_ offers unique advantages such as (near)ambient reaction temperatures and potential lower energy consumption.^44^ MOFs, with their ability to integrate different catalytic sites—ranging from light-harvesting elements (photosensitizers) to reaction centers (catalytic sites) into a single framework, have emerged as excellent candidates for photocatalysis. These assembled composites, using the MOF as a platform host to multicomponent active sites, can serve as catalysts at the solid–gas and solid–liquid interfaces and their porous nature is particularly advantageous in promoting CH_4_ adsorption, thus reducing the reaction pressure compared with conventional thermal processes.

**Figure 6:**
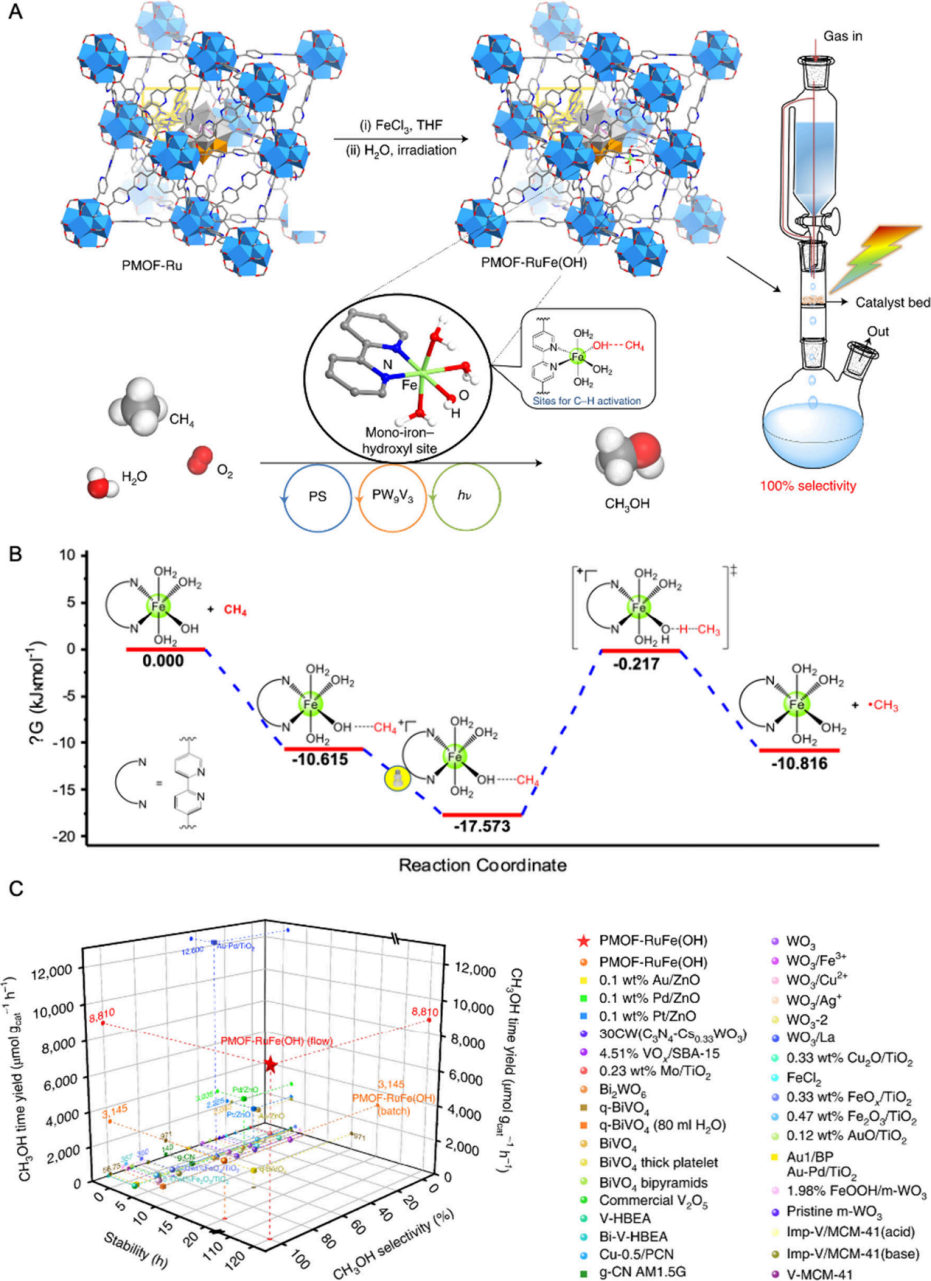
Photocatalytic oxidation of CH_4_ over PMOF-RuFe(OH). (A) Design and synthesis of the PMOF-RuFe(OH) catalyst and the flow reactor for photo-oxidation of CH_4_ to CH_3_OH. (B) Calculated energy profiles for the activation of C–H bond in CH_4_ over confined monoiron hydroxyl sites. Electron-transfer generate the intermediate [(bpy)Fe(OH)(H_2_O)_3_···CH_4_]^2+^ leading to activation of CH_4_. (C) Comparison of the catalytic activity for photo-oxidation of CH_4_ to CH_3_OH over PMOF-RuFe(OH) with other photocatalyst. Reproduced with permission from ref (12). Copyright 2022 The Author(s), under exclusive license to Springer Nature Limited.

With this approach in mind, our group has developed a novel MOF system, PMOF-RuFe(OH), for the efficient photo-oxidation of CH_4_ to CH_3_OH (Figure 6).^12^ The framework is strategically designed with an array of functional elements, including a photosensitizer [Ru(bpy)_3_]^2+^, vanadium-substituted polyoxometalates (PW_9_V_3_), and monoiron hydroxyl sites, positioned within the porous structure. This enables photocatalytic CH_4_ oxidation to CH_3_OH with near 100% selectivity in the presence of O_2_ and H_2_O under illumination of visible light at ambient conditions. The study identifies the binding sites for CH_4_ in the PMOF-RuFe(OH) system, confirming that the mono-Fe(III)-OH species acts as the primary binding site. This is evidenced by strong CH_4_ adsorption and notable hysteretic desorption. *Operando* INS spectra and DFT calculations reveal that CH_4_ molecules interact strongly with the MOF through hydrogen bonding to the Fe–OH groups. The formation of an [Fe–OH···CH_4_] intermediate at the confined monoiron hydroxyl sites effectively lowers the activation barrier for C–H bond cleavage (Figure 6B). We also developed a flow system where a stream of water saturated with CH_4_ and O_2_ flows over a fixed-bed of MOF particles under illumination. Such a process achieves a methane-to-methanol conversion rate (8.81 ± 0.34 mmol g_cat_^–1^ h^–1^) that exceeds that of methane monooxygenase enzymes (5.05 mmol g_cat_^–1^ h^–1^) in nature. Furthermore, this solid catalyst exhibits remarkable durability, maintaining its catalytic activity over 10 cycles or 200 h of continuous operation with little degradation in performance. This catalyst has demonstrated the excellent capacity of MOF materials to integrate multifunctional components into a single structure to facilitate photocatalytic conversion of CH_4_ under flow conditions, and will also inspire the design of catalysts for other multiphasic photochemical reactions. Photocatalytic conversion of CH_4_ to CH_3_OH using MOFs is still at an early stage of development, and many new breakthroughs based upon functional MOF catalysts are yet to be uncovered.

#### 3.2.3 Electrochemical Oxidation of CH_4_

With the increasing sophistication and generation of sustainable green power technologies and the establishment of carbon neutral power grids, the use of electricity to drive chemical reactions under mild conditions is becoming an important area of research. Electrochemistry serves as an effective method to control redox reactions by applying exact electrical biases, providing a cost-effective alternative to traditional chemical processes through electrochemical oxidation. MOFs have also been studied extensively as electrocatalysts for various reactions such as hydrogen evolution reaction (HER), oxygen evolution reaction (OER), oxygen reduction reaction (ORR), nitrogen reduction reaction (NRR), CO_2_ reduction reaction (CO_2_RR), and organic transformations.^45−47^ In contrast, examples of CH_4_ oxidation are rather limited. This is because electrochemical activation of CH_4_ suffers from poor kinetics due to low CH_4_ solubility, although conversion is thermodynamically favorable at modest potentials [*e.g*., CH_4_ (g) + H_2_O (l) → CH_3_OH (aq) + 2H^+^ + 2e^–^ has an equilibrium potential of 0.586 V vs normal hydrogen electrode (NHE) at pH = 0 and 298 K].^48^ This challenge can be overcome by running the reaction at high overpotentials. However, at high overpotentials (especially at ≥0.8 V vs RHE, reversible hydrogen electrode), ORR from water oxidation becomes dominant and occurs at a high rate. This leads to additional energy consumption and significantly increases the cost of CH_4_ oxidation, resulting in low carbon and energy efficiencies. On the other hand, electrochemical processes allow the activation of CH_4_ at room temperature through either the oxygen sites on electrode surfaces or *via* free radicals formed at the electrode/electrolyte interface.^49^ However, the selective production of a specific product in these processes is challenging due to the close electrode potential values of different products and the high reactivity of free radicals and reactive oxygen species. In addition, the relatively low electrical conductivity and limited stability of conventional MOFs under oxidative conditions are key barriers to this field. Thus, the development of novel MOF candidates for electrocatalytic CH_4_OR applications remains a significant challenge, particularly for the control of selectivity to a single product.

### 3.3 Dry Reforming of Methane (DRM)

Dry reforming of methane (DRM) is one of the most promising chemical processes that can convert CH_4_ and CO_2_ into syngas (a mixture of H_2_ and CO) and/or multiple hydrocarbon products.^50^ The challenge resides in the development of catalysts that can operate effectively while being resistant to carbon deposition and sintering, two key issues that degrade catalyst performance over time. Compared with conventional DRM systems at high temperature, MOF catalysts can operate at low temperatures primarily due to their highly tunable structures, confinement effect, active metal nodes, functionalized moieties, and unique redox regulation capabilities, overcoming the limitations by traditional catalysts in DRM reactions. For example, Fan and co-workers successfully developed a plasma-assisted DRM system using Pt nanoparticles doped MOF catalyst (PtNP@UiO-67).^51^ With the activation of nonthermal plasma (NTP), CH_4_ and CO_2_ were converted directly into H_2_, CO, C_2_ and C_3_ hydrocarbons over PtNP@UiO-67 at room temperature. The highly dispersed active sites and tunability of the MOF structures can reduce carbon deposition, thus enhancing the stability of DRM reactions. However, kinetic constraints restrict MOFs from being efficient catalysts for DRM under low-temperature conditions. Therefore, future research requires the optimization of the structure and functionality of MOFs to achieve more efficient CH_4_ conversion at low temperatures.

### 3.4 Nonoxidative Coupling of Methane (NOCM)

**Figure 7:**
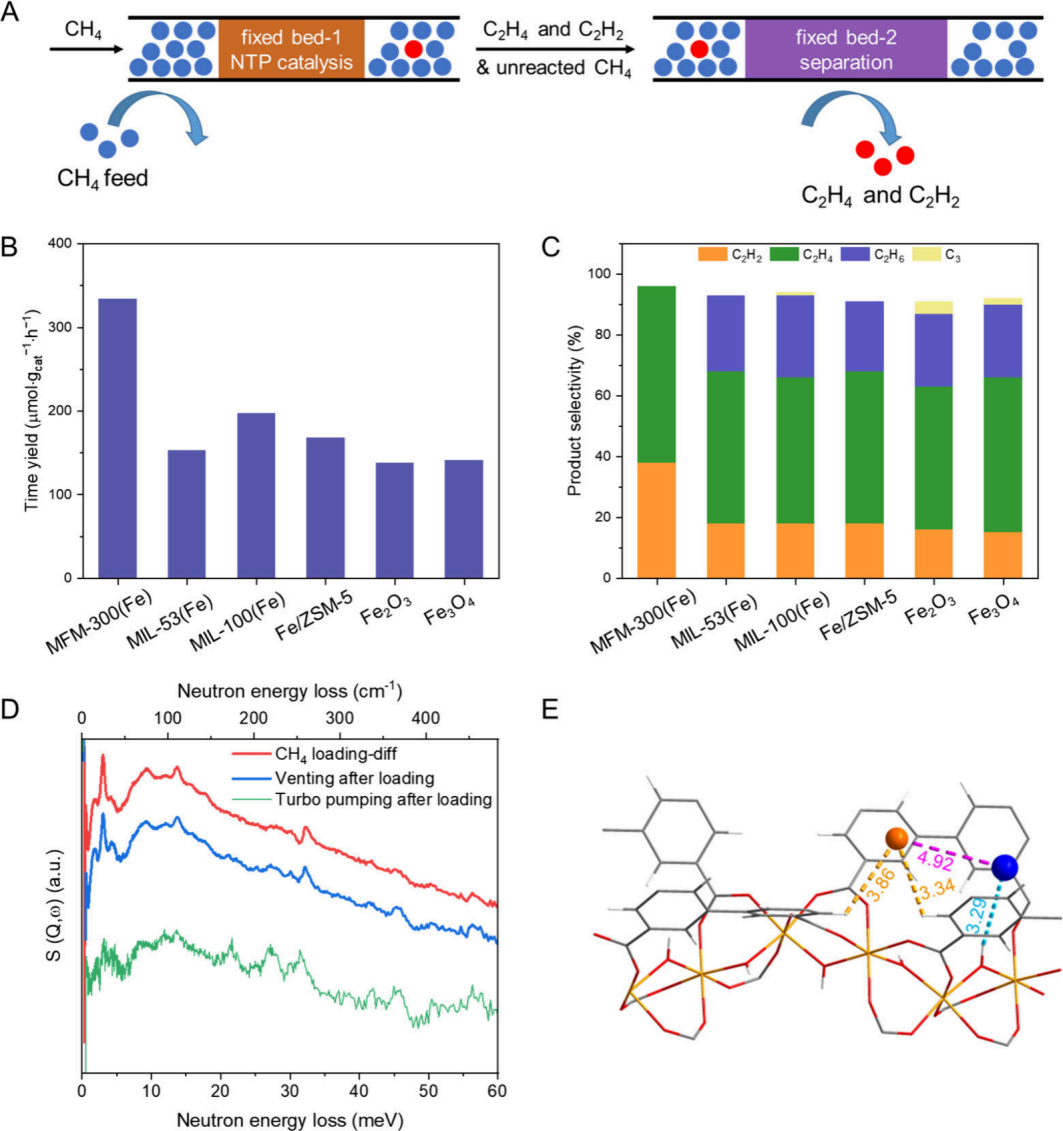
Nonoxidative coupling of CH_4_ over MFM-300(Fe). (A) Scheme of the cascade system integrating the processes of CH_4_ catalysis and product separation. (B,C) Catalytic activity for CH_4_ conversion and selectivity for C_2+_ products. (D) INS spectra for CH_4_-dosed MFM-300(Fe) and that upon desorption under dynamic vacuum. (E) Crystal structures of CD_4_-loaded MFM-300(Fe) and views of the binding sites for CD_4_ molecules in MFM-300(Fe). The structure was derived from Rietveld refinement of NPD data collected at 7 K (C, gray; O, red; Fe, light orange; H, white; for CD_4_ molecules, site I, blue; site II, orange). Reproduced with permission from ref (52). Copyright 2023 The Authors.

Nonoxidative coupling of methane (NOCM) is a promising route to produce value-added higher hydrocarbons (C_2+_) from natural gas. Compared with oxidative coupling of methane (OCM), NOCM processes do not rely upon the use of oxidants, thus effectively preventing the overoxidation of C_2+_ products to CO_2_ and potentially promoting greater product selectivity. Recently, we have reported the direct conversion of CH_4_ to ethylene (C_2_H_4_) and acetylene (C_2_H_2_) driven by NTP under ambient (room temperature and 1 atm) and flow conditions over a MOF material, MFM-300(Fe) (Figure 7).^52^ A high time yield of 334 μmol·g_cat_^–1^·h^–1^ was achieved for C_2_H_4_ and C_2_H_2_, with the combined selectivity reaching 96%. In addition, the time yield and selectivity to C_2+_ hydrocarbons exceeded 2056 μmol·g_cat_^–1^·h^–1^ and 98% at a high CH_4_ conversion of 10%, which compares favorably with state-of-the-art catalytic systems. By combining complementary experimental and modeling techniques, including NPD, INS and DFT calculations, we find that the binding of CH_4_ molecules to the [Fe–O(H)–Fe] sites within MFM-300(Fe) is crucial to the activation of the C–H bond. *In situ* electron paramagnetic resonance (EPR), solid-state nuclear magnetic resonance (ssNMR) and diffuse reflectance infrared Fourier transform (DRIFT) spectroscopies have unravelled the important role of Fe–O(H)–Fe sites in stabilizing reaction intermediates, forming [Fe–O(CH_3_)–Fe] related adducts and achieving a highly selective and efficient NOCM process. Importantly, online separation of C_2_H_4_ and C_2_H_2_ from unreacted CH_4_ was realized *via* a cascade fixed-bed system using benchmark sorbent materials. This work integrates the processes of highly efficient and selective CH_4_ conversion, ease of product separation, and ambient operating conditions, offering a promising route to the production of value-added chemicals from abundant natural gas.

### 3.5 Borylation of CH_4_

Borylation of CH_4_ with bis(pinacolborane) (B_2_pin_2_) has been tested as a promising strategy for activating and converting CH_4_ into value-added chemicals and fuel products.^53^ However, it remains a challenging task to achieve high selectivity for monofunctionalized products due to the higher reactivity of the monoborylated product CH_3_Bpin than CH_4_. MOF materials adopt well-defined structures, versatile structural diversity and highly tunable pore sizes, enabling the fabrication of MOF-based platforms incorporating active sites for shape-selective catalysis.^54−57^ Due to the difference in sizes and shapes of monoborylated methane (CH_3_Bpin, 0.73 × 0.88 nm, linear) and diborylated methane (CH_2_Bpin_2_, 0.80 × 1.39 nm, bent), a potential route exits to selectively convert and separate CH_4_-borylated products *via* shape-selective catalysis. The unique shape-selectivity of MOF-based catalysts originates from their hierarchical pores and highly accessible low-coordinated single metal sites. These provide a promising route for the activation and conversion of inert CH_4_ molecules into fine chemicals.

## 4 Conclusion and Outlook

This Account summarizes state-of-the-art CH_4_ conversion systems using MOF materials. The diverse and tunable active sites within MOF materials enable the direct activation and conversion of the inert CH_4_ molecules into value-added chemicals *via* various routes, including oxidation of CH_4_ to alcohols, dry reforming of CH_4_, nonoxidative coupling of CH_4_, and borylation of CH_4_. MOF-based materials have shown several advantages in CH_4_ catalysis:

(i) Low-coordinated active metal sites, which are inaccessible in homogeneous phases, can be incorporated and stabilized within the MOF frameworks, effectively lowering the activation barrier of CH_4_ molecules.
(ii) The well-defined and ordered structures of MOFs allow the control and identification of active sites at atomic precision by advanced diffraction, scattering and spectroscopic techniques, and the elucidation and visualization active sites for CH_4_ activation and conversion at a molecular level.
(iii) The high porosity and tunable pore sizes of MOFs make the active sites highly accessible for reactants, and the open channels in MOFs allow the transport of the reactants and products, especially under flow conditions, greatly enhancing catalytic activity and selectivity.
(iv) The shape selectivity resulting from the robust and hierarchical porosity of MOFs facilitates the formation of desired products while preventing the overoxidation of CH_4_ molecules.
(v) MOF-derived materials, such as porous carbons and metal-based materials (usually obtained *via* calcination of MOFs), combine the advantages of both the parent MOFs (*e.g*., high porosity and tunable pore size) and the composite materials (*e.g*., high electrical conductivity and high chemical stability), exhibiting unprecedented catalytic activity and stability.

Despite the above advantages, CH_4_ activation and conversion using MOFs are still at an early stage, but there is undoubtedly significant scope for further progress. Total proven reserves of oil and gas are currently some 6952 Tm^3^, and total reserves of shale gas and coal bed CH_4_ are 214 and 201 Tm^3^, respectively. There is thus huge capacity to produce and utilize CH_4_ within the carbon economy, as long as conversion of CH_4_ to green-house CO_2_ is inhibited. However, the CH_4_ in permafrost hydrates is considered a “frozen” source of energy, and geophysical monitoring and modeling are key to predicting the potential release of CH_4_. Some projects have focused on controlled extraction, but commercial-scale production is still far from being realized due to technical challenges in extraction and environmental concerns. CH_4_ remains an important feedstock within the carbon economy and MOFs and MOF-derived materials with high thermal and chemical stability need to be designed and prepared for catalytic CH_4_ conversion. Large-scale production of some benchmark MOFs has been achieved,^58^ and it is entirely expected that more MOF-based materials with specific active sites (*e.g*., single atom metal sites, dual atom metal sites, metal nanoclusters and metal nanoparticles) and pore sizes and functionality will be produced on a large scale, at low cost to facilitate their industrial developments. The underlying reaction mechanisms for CH_4_ catalysis have yet to be thoroughly investigated using state-of-the-art characterization methods with improved time/space resolution. Understanding the interactions between CH_4_ molecules and MOF materials can further guide the design and synthesis of new efficient and selective catalysts. In addition to catalysts, large-scale operations for direct CH_4_ conversion remain a significant challenge. Central to this challenge is the low reactivity of CH_4_ under conditions that can also facilitate product recovery, where new efficient recovery methods must be developed. MOF materials can aid in this area by acting as dual catalyst-separation platforms. Dilute aqueous products are not attractive and much higher product concentrations are required to aid product recovery. Such concerns exist for CH_4_ conversion over both MOF catalysts and other catalysts. Finally, the integration of MOF materials with innovative catalytic methodologies (*e.g*., photocatalysis, electrocatalysis and NTP catalysis) is a promising strategy to achieve new pathways for CH_4_ activation and conversion. However, these techniques are more complex than conventional thermocatalytic reactors and require the consideration of additional factors. For example, in photocatalysis, photocatalysts must also take into account light absorption, charge carrier migration, and various photogenerated active species and intermediates. The design of the photoreactor needs to consider the light source configuration (light propagation and distribution) and the characteristics of the light source (*e.g*., intensity, frequency, monochromaticity, polarization) to maximize the use of solar energy. These considerations are challenging for both laboratory research and industrial applications. Thus, with increasing interest and persistent efforts from both researchers and industry, the development of new efficient and selective CH_4_ catalytic systems based on MOF and related composite materials is to be expected.

## Supporting Information Available

Further introduction of NPD and INS techniques, additional discussions of MOFs in selective methane conversion, and additional schemes showing different pathways for CH_4_ activation (PDF)
