# Supplementary material for: Activation and Catalysis of Methane over Metal–Organic Framework Materials
Source: Acc Mater Res. 2024 Nov 7;6(1):77–88. doi: 10.1021/accountsmr.4c00279 (PMC11773449; doi:10.1021/accountsmr.4c00279)
Supplement: Supplementary file 1 — mr4c00279_si_001.pdf [file mr4c00279_si_001.pdf]

Supporting Information

Activation and Catalysis of Methane over Metal-Organic Framework Materials

Bing An,[1#] Yujie Ma,[1#] Xue Han,[2] Martin Schröder[1]* and Sihai Yang[1,3]*

[1]Department of Chemistry, University of Manchester, Manchester, M13 9PL, UK

[2]College of Chemistry, Beijing Normal University, Beijing 100875, China.

[3]College of Chemistry and Molecular Engineering, Beijing National Laboratory for Molecular Sciences, Peking University, Beijing 100871, China

Table of Contents

Section S1. Neutron powder diffraction and inelastic neutron scattering

Section S2. Summary of methodologies to avoid the over-oxidation in conversion of CH_4 to CH_3OH

Section S3. Features of MOFs in selective methane oxidation and conversion

Section S4. Different pathways for CH_4 activation

References

Section S1. Neutron powder diffraction and inelastic neutron scattering

Neutron powder diffraction (NPD) uses the interaction between neutrons and atomic nuclei to study the structure of materials.[1] Compared with X-ray diffraction, NPD is more sensitive to light elements, such as hydrogen and carbon[2], making it particularly suitable for studying hydrogen-containing compounds. In adsorption studies, NPD provides atomic-level information about the positions and arrangements of adsorbed molecules within crystalline hosts, such as metal-organic frameworks (MOFs), zeolites, helping to elucidate the adsorption mechanism. In catalytic studies, this technique is valuable for identifying structural changes of active sites within the catalysts, offering insights into reaction pathways.

Inelastic Neutron Scattering (INS) measures the energy loss of neutrons after inelastic collisions with materials to investigate molecular vibrations and dynamics.[3] For adsorption and catalysis studies, INS is powerful for probing the interactions between adsorbed molecules and the MOF framework, revealing vibrational states and activation mechanisms. This is particularly valuable for studying the activation of small molecules over porous materials, such as MOFs. Direct visualisation of the interaction between adsorbed CH_4 and the active sites is crucial to understanding the molecular details of adsorption and activation of CH_4 into CH_3OH. INS is a powerful neutron spectroscopy technique to investigate the dynamics (particularly for H-containing compounds, such as hydrocarbons) of host-guest interactions because it has several unique advantages[4]:

- INS spectroscopy is ultra-sensitive to the vibrations of hydrogen atoms, and hydrogen is ten times more visible than other elements due to its high neutron cross-section.
- The technique is not subject to any optical selection rules. All vibrations are active and, in principle, measurable.
- INS observations are not restricted to the centre of the Brillouin zone (gamma point) as is the case for optical techniques.
- INS spectra can be readily and accurately modelled: the intensities are proportional to the concentration of elements in the sample and their cross-sections, and the measured INS intensities relate straightforwardly to the associated displacements of the scattering atom. Treatment of background correction is also straightforward.
- Neutrons penetrate deeply into materials and pass readily through the walls of metal containers making neutrons ideal to measure bulk properties of this material.
- INS spectrometers cover the whole range of the molecular vibrational spectrum, 0-500 meV (0-4000 cm^{-1}).
- INS data can be collected at low temperature (at 5 K in this case), where the thermal motion of the MOF material and the adsorbed CH_4 molecules is significantly reduced.
- Calculation of the INS spectra by DFT vibrational analysis can be readily achieved, and DFT calculations relate directly to the INS spectra, and, in the case of solid-state calculations, there are no approximations other than the use of DFT eigenvectors and eigenvalues to determine the spectral intensities.

Section S2. Summary of methodologies to avoid the over-oxidation in conversion of CH_4 to CH_3OH

For the selective oxidation of CH_4 to CH_3OH, avoiding the over-oxidation of CH_3OH to CO_2 or other by-products is a key challenge. Various strategies have been applied across different catalytic systems to enhance the selectivity of CH_3OH and minimise over-oxidation into by-products:

1. Controlling reaction temperature

- Low-temperature reaction: Lower reaction temperatures suppress the further oxidation of CH_3OH to formic acid or CO_2. By controlling the temperature, CH_3OH selectivity can be maximised, while avoiding excessive oxidation of CH_4.

- Temperature-sensitive catalysts: Using catalysts that are sensitive to temperature allows precise regulation of the reaction conditions, reducing the over-oxidation.

2. Using selective oxidants
   - Mild oxidants: Using mild oxidants such as H_2O_2 or nitrites can effectively avoid the over-oxidation compared with strong oxidants such as O_2.
   - Stepwise addition of oxidants: Gradually adding oxidants into the reaction system ensures controlled reaction progression, preventing the further oxidation of CH_3OH.
3. Regulating reaction intermediates
   - Radical inhibitors: Selective oxidation of CH_4 often involves radical processes. Introducing radical inhibitors can control the concentration of radicals, preventing CH_3OH from undergoing further oxidation.
   - Reaction pathway control: By using appropriate reaction conditions and catalysts, the oxidation pathway can be terminated at CH_3OH formation rather than proceeding to over-oxidation.
4. Introducing reductive atmosphere
   - CO co-feeding: Introducing CO into the reaction system can act as a reducing agent, suppressing deep oxidation. CO reacts with reactive oxygen species, preventing them from further oxidising CH_3OH.
   - Hydrogen assistance: A small amount of H_2 in the reaction system can also effectively inhibit over-oxidation by reacting with reactive oxygen species.
5. Solvent effects
   - Hydration effects: In some catalytic systems, solvents, such as water, can regulate the reaction environment by creating hydration effects that prevent further oxidation of CH_3OH.
   - Non-polar solvents: Selected non-polar solvents can reduce the interaction between oxidants and CH_3OH, preventing further oxidation.
6. Controlling oxygen concentration
   - Limited oxygen supply: Strictly controlling the oxygen concentration in the reaction system can prevent the over-oxidation of CH_3OH. Techniques such as staged oxygen supply or using low-concentration oxygen sources can be employed.
7. Optimising reaction time
   - Short reaction time: Reducing the reaction time limits the contact between CH_4 and oxidants, preventing side reactions.
   - Flash oxidation techniques: Utilising pulse oxidation or other flash reaction techniques allows high-selectivity oxidation in a very short time, avoiding prolonged reactions that lead to over-oxidation.

Section S3. Features of MOFs in selective methane oxidation and conversion

MOFs show several advantages in the activation of CH_4:

1. High surface areas and porous structures

MOFs show high surface areas and tunable pore sizes, which facilitate effective adsorption of CH_4 and O_2.

2. Unique molecular sieving effect

The porous structure of MOFs can provide “molecular sieving effect,” where methanol can rapidly desorb from the active sites upon formation, preventing further reaction with reactive oxygen species, and thus avoiding over-oxidation. This structural design can significantly improve methanol selectivity.

3. Multifunctionality and synergistic effects

The synergy between the organic linkers and inorganic metal nodes in MOFs often enhances catalytic selectivity. Additionally, MOFs can be combined with other catalytic materials to further optimise the selectivity of methane oxidation reactions. The presence of coordinatively unsaturated metal sites in many MOFs offers unique active centers for methane activation. These catalytic sites can activate the C-H bonds in methane. Furthermore,

MOFs can be functionalised with hydrophobic or hydrophilic groups. This flexibility allows MOFs to create an environment conducive to methane adsorption and activation.

4. Design flexibility

The structure and composition of MOFs can be tailored to suit specific applications. Different metal centers, linkers, and pore structures can be optimised for various reaction conditions to minimise methanol over-oxidation. This tunability allows researchers to design MOFs with specific pore sizes, shapes, and surface functionalities, making them ideal for accommodating and activating methane as well as other gas molecules. For instance, modifying pore size or introducing reductive linkers can adjust the desorption rate of methanol, preventing further oxidation.

5. Defect engineering

MOFs allow for defect engineering, where controlled defects can be introduced to enhance catalytic performance, particularly in activating methane. These defects can increase the number of active sites or create pathways that enhance diffusion and adsorption.

To date, challenges, such as thermal stability, stability of active sites, and catalyst recyclability, of MOF-based systems need to be further improved to maximise their potential in selective oxidation reactions.

Section S4. Different pathways for CH_4 activation

Four pathways, namely Shilov cycle, σ-bond metathesis, oxidative addition, and 1,2-addition, are widely proposed in MOF-based catalysts for methane activation.

1. Shilov cycle

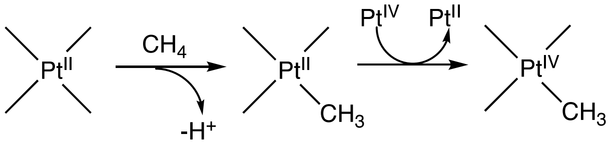

The process has three main steps: (1) C-H activation, (2) a redox reaction to form an octahedral intermediate, followed by (3) the formation of the carbon-oxygen bond to form methanol. This is often observed in Pt-containing MOF catalysts.

2. σ-bond metathesis

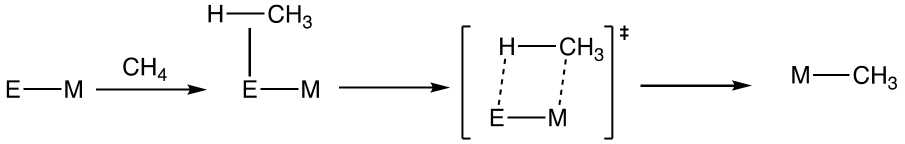

σ-bond metathesis involves the formation of new C-H and metal-carbon bonds, where the active sites are typically in the d^0 configuration in MOF catalysts, e.g., Ce-UiO-Co(OH) catalyst[5].

3. Oxidative addition

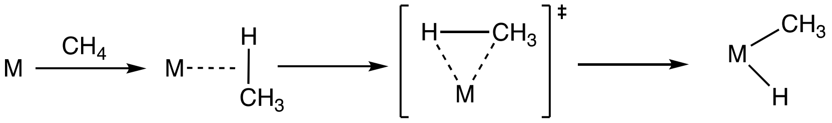

The metal centre coordinates with a σ C-H bond to form an intermediate denoted as σ-methane complex.

4. 1,2-addition

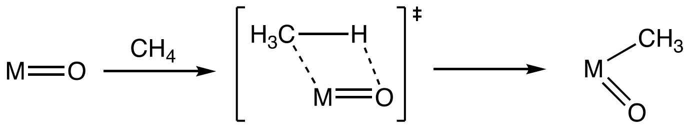

1,2 addition reaction goes through a four-membered transition state. However, a polarised double or triple metal-ligand bond is required to favour the formation of the product, as can be seen in many enzyme-like systems with Cu=O and Fe=O bond in the MOF-based catalysts.

References

(1) Fitch, A. N.; Jobic, H. Structural Information from Neutron Diffraction. In *Structures and Structure Determination*; Baerlocher, C., McCusker, L. B., Olson, D. H., Eds.; Springer: Berlin, Heidelberg, 2001; pp 31-70.
(2) Yan, Y.; Telepeni, I.; Yang, S.; Lin, X.; Kockelmann, W.; Dailly, A.; Blake, A. J.; Lewis, W.; Walker, G. S.; Allan, D. R. Metal–organic Polyhedral Frameworks: High H_2 Adsorption Capacities and Neutron Powder Diffraction Studies. *J. Am. Chem. Soc.* **2010,** *132,* 4092-4094.
(3) Lin, L.; Mei, Q.; Han, X.; Parker, S. F.; Yang, S. Investigations of Hydrocarbon Species on Solid Catalysts by Inelastic Neutron Scattering. *Top. Catal.* **2021,** *64,* 593-602.
(4) An, B.; Li, Z.; Wang, Z.; Zeng, X.; Han, X.; Cheng, Y.; Sheveleva, A. M.; Zhang, Z.; Tuna, F.; McInnes, E. J. L.; Frogley, M. D.; Ramirez-Cuesta, A. J.; L, S. N.; Wang, C.; Lin, W.; Yang, S.; Schröder, M. Direct Photo-Oxidation of Methane to Methanol over a Mono-Iron Hydroxyl Site. *Nat. Mater.* **2022**, *21*, 932-938.
(5) Antil, N.; Chauhan, M.; Akhtar, N.; Newar, R.; Begum, W.; Malik, J.; Manna, K. Metal–Organic Framework-Encaged Monomeric Cobalt(III) Hydroperoxides Enable Chemoselective Methane Oxidation to Methanol. *ACS Catal.* **2022,** *12,* 11159-11168.
